# Facebook as a Learning Tool: Perception of Stroke Unit Nurses in a Tertiary Care Hospital in Islamabad

**DOI:** 10.7759/cureus.2357

**Published:** 2018-03-22

**Authors:** Maimoona Siddiqui, Ahmed S Bukhari, Ibrahim Shamael, Zubana A Shah, Neil Maken

**Affiliations:** 1 Consultant Neurologist, Department of Neurology, Shifa International Hospital, Islamabad, Pakistan; 2 Research Associate, Department of Neurology, Shifa International Hospital, Islamabad, Pakistan; 3 Shifa Institure of Medical Technoloy, Shifa International Hospital; 4 SCMT, Shifa International Hospital, Islamabad, Pakistan

**Keywords:** facebook, blended learning, stroke course, stroke unit nurse, islamabad, pakistan, asia

## Abstract

Objective

To obtain the perception of nurses on the use of Facebook as a learning tool.

Materials & methods

We conducted a pilot observational study in which data were collected through a detailed course evaluation and feedback survey questionnaire. Twelve stroke care nurses were enrolled in a stroke course specifically designed to provide participants with information and knowledge about stroke unit nursing care. Firstly, a closed Facebook group consisting of the participants and facilitators was created. An activity in accordance with the course content was posted in the group daily. Before the start of the course, a pre-course test was conducted. The four-week course culminated in a graded written examination. Its results were compared with the pre-course test. A detailed feedback questionnaire was given to the participants at the end of the course, which was specifically designed to elicit perceptions of nurses about the use of Facebook as a learning tool.

Results

Of the 12 enrolled nurses, 10 completed the certification and the post-course feedback evaluation. Facebook was used by all participants as a platform to view and study the course contents. The timing of the course activities was rated “very good” by three and ‘good’ by six of 10 participants. However, one of the major issues faced by five participants was problematic internet access. The overall rating of the course was “very good” by five participants, “good” by three, and “satisfactory” by two of 10 participants. The post-course test showed that nine of 10 candidates passed with scores >70% compared to only two candidates getting scores >50% in the pre-course test.

Conclusion

Facebook use enabled participants to study the material when their schedule permitted them. The online teaching and facilitation were ideal for our full-time stroke unit nurses as reflected by their improved post-course test results.

## Introduction

The use of social networking technology in medical education is of growing interest to academics as a potential teaching and learning tool [1].

Shifa International Hospital (SIH) has a stroke unit in operation since 2015. It consists of a multidisciplinary team, with nursing staff as an integral component. The role of a stroke unit nurse is new to the Pakistani healthcare system, but it is well established in developed countries with high patient and staff satisfaction [2]. Even though a dedicated stroke unit at SIH has helped improve patient care, there is always room for more improvement. We at SIH feel that continuous improvement and training for better healthcare provision is mandatory. Short certification courses are helpful in achieving this improvement. Our stroke unit nurses found it difficult to get leave from work and attend a specific course. This gave us an opportunity to explore and use the concept of “blended learning” [3]. This term appears to have been in use since the popular advent of the Internet in the late 1990s [4].

Blended learning is an educational program in which a student learns at least in part through the delivery of content and instruction via online digital media with traditional classroom methods. It requires the physical presence of both teacher and student, with some element of student control over time, place, path or pace [5]. The younger generation of today expects that technology should be an integral part of their education. Social media, including Facebook, is now part of many people’s everyday lives. Facebook is currently the most popular social media platform in America [6]. Facebook is one of the most popular social networks in Pakistan as well, with 30 million registered users [7].

Facebook has been found to increase collaboration between students and increase the communication between students and lecturers [8-10]. It has also helped increase students’ interest in the subject matter [11]. Its inclusion in medical curricula has shown a positive impact in the education of medical students [12].

The objective of this study was to obtain the perception of nurses on the use of Facebook as a learning tool.

## Materials and methods

We conducted a pilot observational study in which data were collected through a detailed course evaluation and feedback survey questionnaire. The content of the questionnaire focused on the blended learning experience. All nurses who participated granted informed consent. They were guaranteed that their responses to the evaluation questionnaire would not affect the evaluation of their performance in the stroke course. The results of the course examination were delivered to the participants before data analysis. Additionally, the course evaluation survey was conducted anonymously. Ethical approval for this study was received from the SIH Institutional Review Board and Ethics Committee.

Participants

Twelve stroke care nurses were enrolled. Participants were eligible for inclusion in the study if they were registered nurses, full-time employees of SIH with at least one year of work experience, had a Facebook account, were able to use computers and smartphones, and completed the full stroke course along with the post-course feedback survey questionnaire.

Facilitators

Facilitators consisted of a neurology department consultant, neurology residents, and physiotherapists of SIH.

Course director

A course director was appointed for the smooth implementation of the course content, provision of classroom facilities, and monitoring of evaluation and feedback.

Course objectives

The course was specifically designed to provide participants with information and knowledge about stroke unit nursing care and the application of that knowledge in the provision of care to patients based on their individual needs.

Course content/activity

The course duration was of four weeks. Firstly, a closed Facebook group consisting of the participants and facilitators was created. All participants and facilitators were instructed to use the group for course-related activities only. The participants were given reading material and internet resources for discussion. An activity in accordance with the course content was posted in the group daily. Participants were encouraged to comment and discuss the topic online and interact with the other members of the group (Table 1).

**Table 1:** Course content and activities. NIHSS: National Institute of Health Stroke Scale

Serial number	Learning Objectives	Teaching/Learning Activities
1	Basic anatomy and physiology of central nervous system and peripheral nervous system	Interactive lecture and online reading material
2	What is stroke? Recognition of stroke, assessment and monitoring of stroke patients, common causes and risk factors of stroke	Interactive lecture and online reading material
3	What is Glasgow Coma Scale (GCS)? Components of GCS Importance of GCS How to Monitor GCS	Interactive lecture, online reading material and online videos
4	Examination of patient including GCS and NIHSS score calculation	Online videos
5	Tissue plasminogen activator (tPA) indications, dosage, and contraindications	Interactive lecture and online reading material
6	Nursing care of stroke patients in first 72 hours	Online reading material and online videos
7	Stroke prevention	Online reading material and online videos
8	Complications after stroke	Online reading material, online videos and a small group activity
9	Principles and strategies in stroke rehabilitation	Online reading material, online videos and a small group activity

Facilitators were responsible for posting the online links to pages of study material and mediating the discussions. Relevant videos were also posted online in the group on a weekly basis.

For online browsing, Safari, Microsoft Internet Explorer, and Google Chrome browsers were recommended. Participants were instructed to use desktop computers, laptops, smartphones, or tablets. They were advised to study the material as per their own convenience. Ninety-minute contact sessions were held once a week in the hospital lecture rooms for interactive lectures, case-based discussions, or role play. During these four weeks, the nurses were to perform their regular duties in the stroke unit.

Evaluation

Before the start of the course, a pre-course test was conducted. After four weeks, the course culminated in a graded written examination. Its results were compared with the pre-course test. A detailed feedback questionnaire was given to the participants at the end of the course, which was specifically designed to elicit perceptions of nurses about the use of Facebook as a learning tool. Responses to the questionnaire were summarized along four parameters (i.e., very good, good, satisfactory, and poor). Participants were also asked to rate different aspects of the course (i.e., the timing of activities, course content, online/small group interactions, clarity of instructions and sequence of activities).

The content included on the questionnaire is shown in Table 2.

**Table 2:** Feedback questionnaire.

Question	Possible Answers
Did you complete the course?	Yes No
Did you find Facebook group helpful in your course?	Yes No
Which browser did you use for viewing online material?	Microsoft Internet Explorer Safari Google Chrome Other
What was your internet connection type?	WIFI LAN 3G/4G
Did you have any problems in accessing the online material?	Yes No If yes please explain (Descriptive Question)
Were you able to see the video portions of the course?	Yes No If no please explain (Descriptive question)
Overall rating of the course	Very Good Good Satisfactory Poor
Rating of components of the course:	
Timing of activities	Very Good Good Satisfactory Poor
Content	Very Good Good Satisfactory Poor
Online and small group interactions	Very Good Good Satisfactory Poor
Clarity of instructions	Very Good Good Satisfactory Poor
Sequence of activities	Very Good Good Satisfactory Poor
Which activity/activities did you find most useful? Why?	Descriptive question
Indicate your experience in the course for the following statements:	
My access to internet to work on the course was sufficient and unproblematic.	Always Frequently Occasionally Rarely
The information received before and during the course was accurate, clear and on time.	Always Frequently Occasionally Rarely
The instructions were clear.	Always Frequently Occasionally Rarely
Overall how would you describe the course in your own words?	Descriptive question
What is your qualification?	Descriptive question
Work experience in years.	Descriptive question

## Results

Of the 12 enrolled nurses, 10 completed the certification and the post-course feedback evaluation. Two participants were excluded as they did not fulfill the inclusion criteria.

Facebook was used by all participants as a platform to view and study the course contents. Eight of 10 participants used a wi-fi internet connection to study, and two used a landline internet connection. Microsoft Internet Explorer and Google Chrome were the two online browsers used to review the online study material. Six of 10 participants were able to streamline the posted video files along with the study material; the other four participants could not access the files.

**Figure 1:**
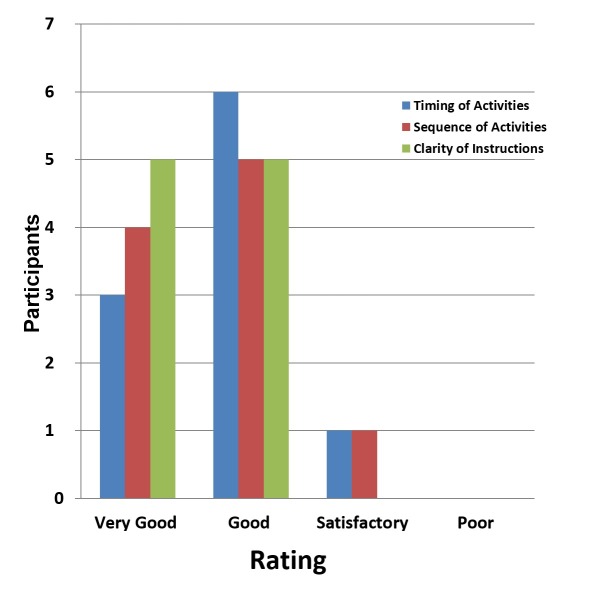
Rating of components of the course.

The timing of the course activities was rated “very good” by three and “good” by six of 10 participants. However, one of the major issues faced by five participants was problematic internet access. The other five participants had very good internet access and internet availability (Figure 1).

**Figure 2:**
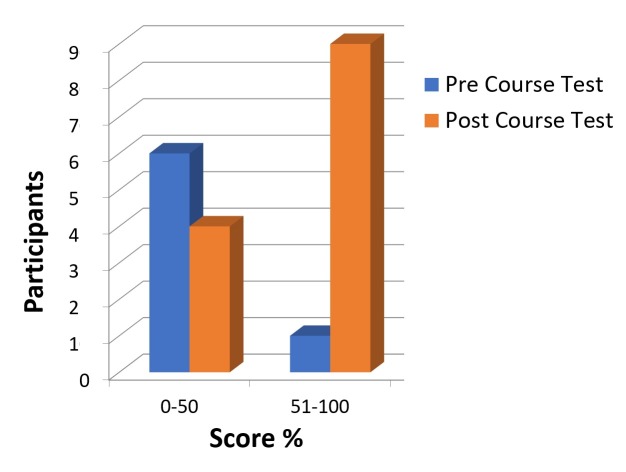
Pre-course and post-course test scores.

Six of 10 participants thought the information received before and during the course was accurate, clear, and on-time. The post-course test showed that nine of 10 candidates passed with scores >70% compared to only two candidates getting scores >50% in the pre-course test (Figure 2).

The overall rating of the course was “very good” by five participants, “good” by three, and “satisfactory” by two of 10 participants. No one ranked the course “poor”.

## Discussion

The overall experience of blended learning was well-received. It does, however, require commitment, activity, and dedication [13]. Providing feasible opportunities to improve knowledge and skills has a positive impact on the technical-scientific competencies that are necessary for workers in the healthcare sector [14].

Participants' preferences should be considered before designing a blended learning course. Appropriate resources, the suitability of course content, and support from the management of the conducting institute are key factors that ensure the success of blended learning [15].

Division of work hours in different shifts and heavy patient loads are factors that were a hindrance in designing a stroke course for nurses using conventional teaching methods.

A major limitation of this study was the limited number of participants. There are still medical personnel who do not use Facebook. Computer literacy among nursing staff is also an important issue to be addressed. Developing knowledge and skills in this area also has the potential to improve staff productivity and raise healthcare standards [16].

A suggested future research proposal is to adopt this research methodology to a large-scale study with a greater number of participants in other medical departments, institutes, and colleges, representing different disciplines and specialties. This will help us get a broader picture of the benefits of using Facebook as a learning tool.

## Conclusions

Facebook enabled participants to study the material when their schedule permitted. The online teaching and facilitation were ideal for our full time working nurses as reflected by their improved post-course test results.
